# Joint Calibration of a Multimodal Sensor System for Autonomous Vehicles

**DOI:** 10.3390/s23125676

**Published:** 2023-06-17

**Authors:** Jon Muhovič, Janez Perš

**Affiliations:** Faculty of Electrical Engineering, University of Ljubljana, Tržaška Cesta 25, SI-1000 Ljubljana, Slovenia; jon.muhovic@gmail.com

**Keywords:** USV, calibration, multimodal system, annotation, autonomous vehicle

## Abstract

Multimodal sensor systems require precise calibration if they are to be used in the field. Due to the difficulty of obtaining the corresponding features from different modalities, the calibration of such systems is an open problem. We present a systematic approach for calibrating a set of cameras with different modalities (RGB, thermal, polarization, and dual-spectrum near infrared) with regard to a LiDAR sensor using a planar calibration target. Firstly, a method for calibrating a single camera with regard to the LiDAR sensor is proposed. The method is usable with any modality, as long as the calibration pattern is detected. A methodology for establishing a parallax-aware pixel mapping between different camera modalities is then presented. Such a mapping can then be used to transfer annotations, features, and results between highly differing camera modalities to facilitate feature extraction and deep detection and segmentation methods.

## 1. Introduction

Multisensor systems are widely used for their versatile array of modalities and their robustness to adverse effects such as poor illumination or weather conditions. Additionally, different modalities can produce richer information that allows easier or higher quality scene understanding. However, the multimodal sensor data can be quite heterogeneous, which makes it difficult to extract the corresponding features from different modalities. This is obvious for point-based sensors such as LiDAR, but even the use of cameras with different modalities can preclude the use of standard methods for image alignment. Yet, since the calibration is necessary for the usability of an autonomous system, the calibration of extrinsic sensor parameters must be performed. Some sensors lend themselves to this problem quite well, but different modalities can prove problematic. Correctly aligning cameras with different fields of view that are not mounted in a stereo fashion or have very different modalities visually is also an especially difficult task that we attempt to solve in this work.

The aim of this work is first to present a robust and simple approach to calibrating a multimodal sensor system that includes multispectral modalities. Furthermore, to facilitate using multimodal data for supervised learning, we propose a method for transferring manual RGB annotations to other modalities or vice versa. This is performed by establishing parallax-aware pixel mappings between image planes, therefore enabling the propagation of arbitrary data between camera images in a well-calibrated system.

## 2. Related Work

LiDAR sensors have proven popular for pairing with cameras, as shown by many published datasets using such a combination. Prime examples of this are the KITTI [1] and Oxford RobotCar [2] datasets. LiDAR measures absolute distances, has long range, and is in most cases less susceptible to lighting conditions than cameras. While methods that utilize point cloud data directly have been proposed, such as PointNet [3] or more recently PointTransformer [4], most approaches still use LiDAR paired with image data. This requires some calibration process to determine the relative positions of the used sensors. Camera–LiDAR calibration has been solved in various different ways in the past, starting with the pioneer work by Zhang et al. [5]. Pandey et al. [6] used a standard calibration checkerboard and used RANSAC to estimate the target plane in LiDAR data. The authors stated that a minimum of three nonplanar views were required for a successful calibration. Guindel et al. [7] used a custom calibration target with features visible for both a camera and LiDAR. The authors described a segmentation algorithm to detect discontinuities on their calibration target in both a stereo and a LiDAR point cloud. Finally, ICP was used to determine the relative sensor positions. The same group extended their approach in [8] by including ArUco markers placed on the calibration target. If the markers can be reliably detected, this approach allows for the calibration of an arbitrary combination of LiDAR and stereo as well as monocular cameras. The authors also performed experiments with LiDAR sensors of different resolutions in synthetic and real environments. Pusztai et al. [9] employed a cardboard box for camera–LiDAR calibration and exploited the unique property of three perpendicular planes to efficiently detect the target in the LiDAR point cloud, but this method still required some points to be manually selected in the camera images. Grammatikopoulos et al. [10] presented a highly reflective planar target that could be easily detected in LiDAR along with a visual marker to establish the image correspondence. Ou et al. [11] presented a highly reflective calibration target that was detectable in both cameras and LiDAR sensors. They also formulated a graph optimization procedure that estimated the extrinsic parameters of the camera–LiDAR system while simultaneously accounting for the inaccuracies of the LiDAR point cloud. Choi et al. [12] proposed a method for calibrating a thermal camera to LiDAR, using an acrylic target with high heat conductivity that was easy to detect in the thermal camera. RANSAC was then used to extract the target position from the LiDAR point cloud, and the EPnP algorithm [13] was used to estimate the extrinsic parameters.

Methods that are able to estimate camera–LiDAR calibration without a specific target object have also been published. Levinson et al. [14] proposed an online method for calibrating LiDAR and a camera by aligning the discontinuities in LiDAR and the edge responses in the camera image. Pandey et al. [15] separately proposed their method of targetless calibration using mutual information. Aside from feature extraction, the calibration is mostly a geometrical problem, but end-to-end deep learning based methods have also been developed. Iyer et al. [16] proposed CalibNet, a deep learning architecture that directly predicted extrinsic parameters from an RGB image and a projection of LiDAR points to a 2D plane. A similar approach was also taken with LCCNet [17] and CFNet [18]. Yuan et al. [19] presented a targetless camera–LiDAR calibration method that worked by aligning the natural edge features in both modalities. The authors proposed using depth-continuous edges as LiDAR features. These were obtained by locally detecting planes using RANSAC. The edges extracted from the LiDAR sensor and the image edges obtained from Canny algorithm were finally used for sensor alignment.

Instead of extracting simple features from image data, Zhu et al. [20] have proposed using rich information from semantic segmentation to serve as image features. The semantic masks can then be used to guide the optimization process instead of simpler image features. Similarly, Wang et al. [21] introduced semantic centroids to match semantic labels from both LiDAR and image data.

The purpose of aligning two cameras is to extract more information from the observed scene. The simplest approach for such a goal is a stereo system. The process for calibrating such a system was first described by Takahashi et al. [22]. Such a system uses two cameras that are offset on a single axis (usually *x* axis) and uses the parallax effect to estimate the pixel distance. However, when using cameras with different modalities, the goal is instead to align the camera images, so their image planes overlap perfectly. Thus, the scene can be identically observed in different modalities. This is only possible in two ways: by splitting the light passing through a single camera lens and directing it to different imaging sensors or by reconstructing the depth of the entire scene. Under any other circumstance, the parallax effect produces different displacements for points at different depths in both cameras, and perfect alignment is not possible.

The beam-splitter approach was described by Hwang et al. [23], where the authors detailed the special hardware configuration used to acquire pixel-aligned multispectral images. The result of this approach was pixel-aligned images that were used to compile a dataset for pedestrian detection in low-light conditions. If the depth information of the observed scene is known, by using a stereo camera system along with a different modality camera, the pixels can be mapped accurately if the cameras are mounted closely together. Rangel et al. [24] used a depth camera together with a thermal camera to implement a thermal–depth fusion pipeline. They also described the difficulties of constructing a calibration target that was easily visible in both the visual and thermal spectrum. Shivakumar et al. [25] similarly combined a stereo RGB camera with a thermal camera to correctly overlap images. The authors used aluminum squares to construct a calibration pattern that was visible in both modalities. A dataset and a CNN architecture for semantic segmentation were also proposed in the same work.

Increasingly larger multimodal datasets are being published, especially for autonomous cars, such as Cityscapes [26], KITTI [1], nuScenes [27] and the Waymo Open Dataset [28]. These contain millions of images and hundreds of thousands of annotated frames, often with 3D bounding boxes for LiDAR data, as well as semantic labels for images. Manually annotating data is time-consuming and expensive at best and almost impossible at worst. Annotating high quality RGB images, while difficult, is possible for human annotators, as is annotating dense LiDAR data. However, when dealing with other modalities such as IR or thermal data, annotation, especially dense annotation, becomes prohibitively hard. The authors of the LLVIP dataset [29] combined RGB with infrared images for low-light pedestrian detection and also explored different methods of fusing the modalities. However, the image registration was performed semi-manually, which included manually selecting the corresponding points on both images.

González et al. [30], the authors of the CVC-14 dataset, used a thermal camera coupled with a visible light camera to detect pedestrians. The authors claimed the small baseline of the cameras made the disparity and occlusions negligible. Lee et al. [31,32] presented the ViViD dataset that combined RGB-D images with RGB images, thermal images, and event camera data, as well as LiDAR data. They used a heated calibration target to calibrate the thermal camera with LiDAR. Alternatively, some works have used high level deep learning approaches to align modalities. Kniaz et al. [33] presented ThermalGAN, a generative model that performed color to thermal image registration for the purposes of person re-identification.

## 3. System

Our sensor system was designed with a focus on capturing data on unmanned surface vehicles, (USVs); so, the choice of sensors reflects that. The system is described in detail in [34]. A diverse array of sensors is useful on or near water, because the visual conditions can be markedly different from the conditions on the ground, with problems such as glare on the water surface, nearly submerged obstacles, different lighting conditions, etc. The presence of water might also adversely affect the usability of stereo cameras, since water can be difficult to reconstruct using stereo methods during periods of extreme calm.

Our sensor system included the following visual sensors:

- Velodyne VLP-16 LiDAR
- Stereolabs ZED 2
- Dual NIR camera
- Teledyne FLIR BFS-U3-51S5P-C polarization camera
- Thermographic (long wave infrared) camera Device-ALab SmartIR384L.

Our Velodyne LiDAR sensor is a 16-beam version of a 64-beam LiDAR that is widely used in automotive sensoring research datasets such as KITTI [1] and Oxford RobotCar [2]. It has a range of 100 m and is capable of producing around 30k points with each rotation. Stereolabs ZED is a stereo camera solution with baseline 12 cm that has a working depth of 20 m and is capable of producing depth point clouds in 2k resolution in real time. Our polarization camera simultaneously captures images through four directed polarization filters. Its purpose is to acquire data that are robust to glare from the water surface. The thermographic camera SmartIR384L we use is responsive to light in the 8–14 μm spectrum and has a viewing angle of 50°.

**Figure 1:**
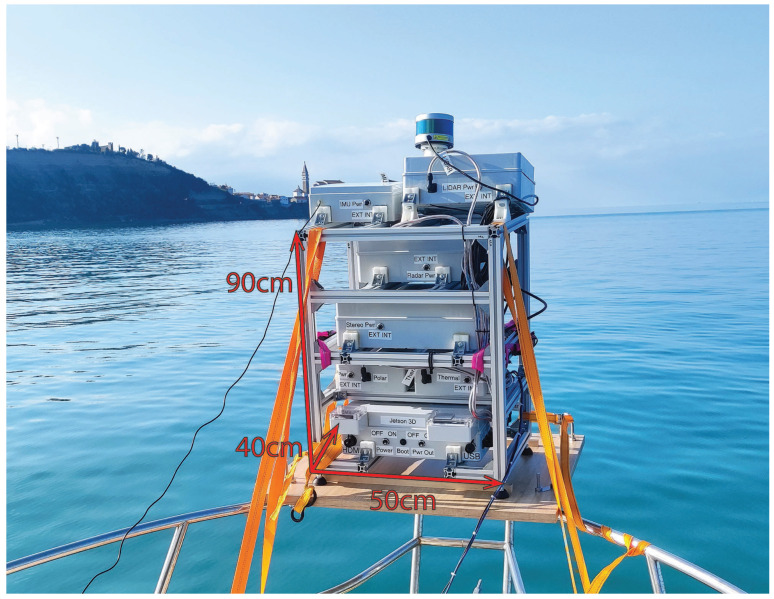
Our sensor system during recording in a marine environment, overlaid with its approximate physical measurements.

The dual near infrared (NIR) spectrum camera was built as a combination of two RaspiCam camera modules without a NIR-blocking filter. One of the camera modules is coupled with the Kodak Wratten #87C NIR low-pass filter with a cutoff wavelength of 900 nm (NIR camera, in text referred to as IR1), and the other is coupled with a band-pass filter with a central wavelength of 975 nm and Full Width-Half Max (FWHM) of 25 nm (in text referred to as IR2). There is an absorption peak for water at 975 nm; therefore, the water appears black, while it appears transparent through the low-pass NIR filter, at least under laboratory conditions. Therefore, the combination of these modalities should be able to provide some information about the presence of water by comparing the aligned image pixels of low pass NIR and 975 nm NIR image. The composition of our sensor system is shown in Figure 1. Due to its modular design, the sensors were not mounted very closely together; therefore, the parallax effect was not negligible.

## 4. Methods

Our methods aim to automatically establish the extrinsic parameters of the cameras and LiDAR sensor in a multimodal sensor system. This is performed by estimating each of the camera positions relative to the LiDAR sensor. Since the camera and LiDAR positions in the system are fixed, the resulting relative positions can be used to establish the relative positions between the cameras themselves. While the procedures for establishing camera–LiDAR calibration have been extensively studied, both with and without using calibration targets, a unified approach for calibrating multimodal systems has thus far not been proposed. Due to the difficulty in extracting the pertinent features from heterogeneous modalities, direct position estimation might be impossible. We present a method for calibrating a system that enables extrinsic calibration even in very different modalities using only a simple planar target. The method is able to detect the target features in any of the used camera modalities, thus a camera–LiDAR extrinsic calibration is made possible. We also present an approach that establishes pixel mapping between cameras in order to produce pixel-aligned multimodal images or to transfer manual annotations to nonannotated images. This can in turn facilitate the use of deep models for multimodal feature extraction, object detection, or semantic segmentation.

The following sections present various parts of our proposed approach. Section 4.1 details the structure and design of the calibration target used in our method. Section 4.2 presents how to establish the relative positions of a camera and a LiDAR sensor, while Section 4.3 explains how to establish pixel mapping between images of different cameras.

### 4.1. Calibration Target

The established approach for calibrating the intrinsic and extrinsic parameters of cameras (e.g., in stereo systems) is to use a known object, referred to as the calibration target. The calibration target is usually a distinct object with an easily detectable pattern or shape. When dealing with different modalities, special care is needed in calibration target design. That is because some sensors might be unable to consistently detect the standard calibration patterns used for visual spectrum cameras. Different designs, materials, and shapes can be used to facilitate detection.

While methods that sidestep the need for a specific calibration object have been proposed in the past, the variety in sensor modalities can preclude such an approach. We thus constructed an asymmetric circle grid target of size 1.05 m ×  1.75 m, with 6 cm black plastic circles spaced 30 cm apart. This makes the target easy to detect for RGB and polarized cameras by using standard methods. IR and thermal cameras, however, have problems detecting the target consistently. The filters on the IR cameras on our system block most of the visible spectrum, which makes the images dark in the absence of very bright conditions and requires higher camera gain, increasing the noise. Additionally, the thermal camera usually cannot detect the calibration pattern because the temperature of the calibration surface is close to uniform. Differences in the emissivity between the black dots and the target itself do not provide enough contrast either.

**Figure 2:**
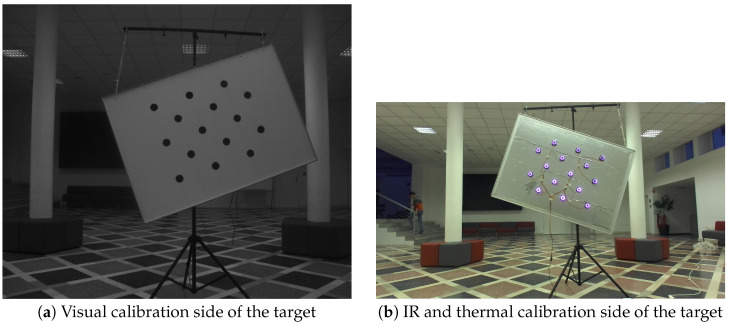
A two-sided planar calibration target used for the calibration of our system.

**Figure 3:**
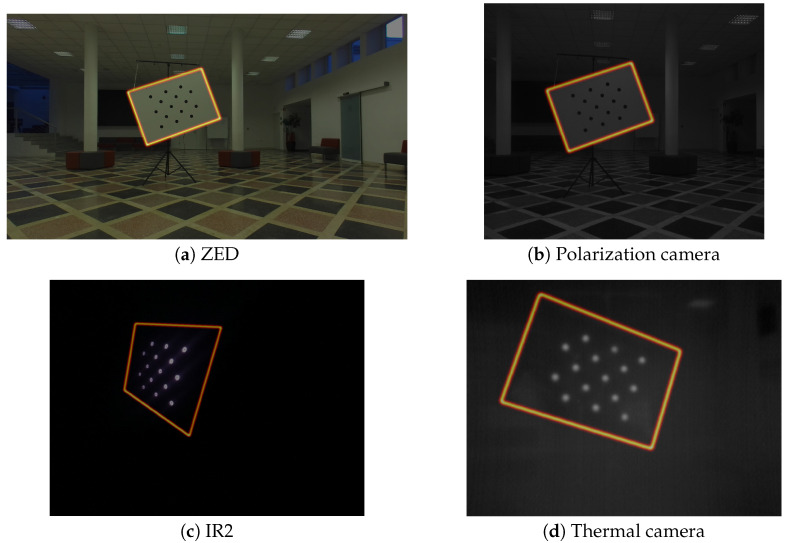
Edges of the calibration target extrapolated from the calibration target model (asymmetric circle grid), shown in different modalities.

We opted to use IR light sources to address the problem of calibration pattern detection for both the IR cameras and the thermal camera. We used a circular array of IR LED diodes for each of the calibration pattern circles on the reverse side of the target, exactly aligned with each circle. This provides bright easy-to-detect dots in both IR cameras, and the temperature difference between the circular LED array and the rest of the target surface enables the pattern detection with the thermal camera. Figure 2 shows how the IR-focused calibration target looks in the visual spectrum, while the bottom row of Figure 3 shows how the other side of the target looks through both the NIR and thermal camera.

Additionally, to enable the detection of the horizontal target edges in the LiDAR point cloud, the calibration target was rotated for about 30° on the *z* axis to produce intersections with multiple LiDAR beams. This allows for the consistent detection of all four edges of the target object in the LiDAR point cloud.

### 4.2. Camera–LiDAR Calibration

Calibrating LiDAR to a camera requires projecting 3D points onto the image plane, which requires the intrinsic parameters of the camera. These can be calculated using the standard method proposed by Zhang [35]. The method requires only the coordinates of the target pattern in their own coordinate system and their corresponding 2D coordinates observed in the image. By using both variants of our calibration target, many images of the calibration target can be obtained for each of the used cameras. Given the size of the target, it can be detected in various positions within the crucial working range of our system (2–20  m). In the course of our investigation, we found that, when using from 50 to 100 images for each camera, the reprojection error of under 1 pixel could be achieved. The resulting intrinsic parameters can then be used both for localizing the target edges (the geometric 2D relation between the circular pattern and target edges is known in advance), as well as for projecting the LiDAR points onto the image plane.

#### 4.2.1. Image Features

**Figure 4:**
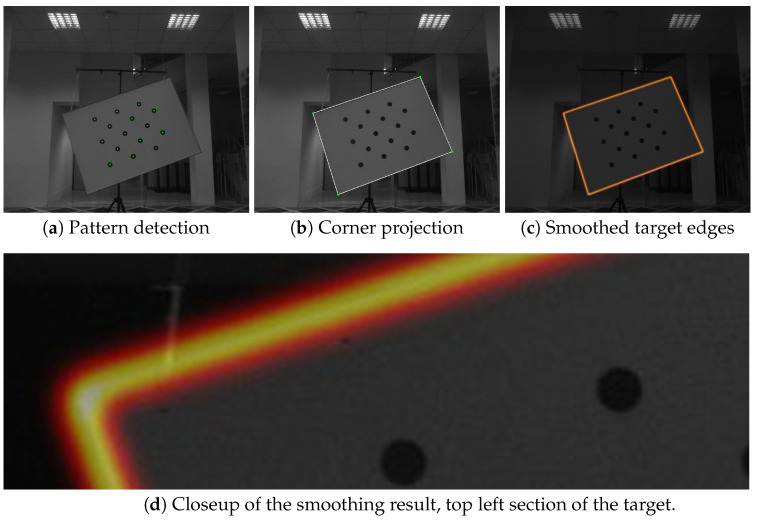
The calibration target-edges detection process. First, the pattern is detected, then the corners are projected to the image, and finally, the edges are smoothed using a Gaussian kernel.

The image features used for aligning the coordinate systems of a camera and the LiDAR sensor were the edges of the target plane (see Figure 3). The calibration pattern can be consistently detected in the image, and because of its known physical size, the relative position of the target and camera coordinate systems can be established using perspective-n-point approaches, which can estimate a physical object’s position relative to camera, if its feature points are observed in the image. When the relative position of the target pattern to the image plane is established, it is trivial to describe target corners’ positions in the target coordinate system and project them onto the image plane. When the lens distortion was removed using intrinsic parameters, the target edges could be localized by connecting the corner projections with straight lines. In order to create a gradient that will facilitate optimization, the edges were smoothed with a Gaussian kernel with its size relative to the camera resolution. We used the kernel width of σ=0.015×imagewidth. The process is depicted in Figure 4. Note that the resulting smoothed band is quite narrow—the optimization starts with pretty good estimates of the relations between the LiDAR and the image due to the known measurements of the sensor positions within the sensor stack, as shown in Figure 1.

#### 4.2.2. LiDAR Features

**Figure 5:**
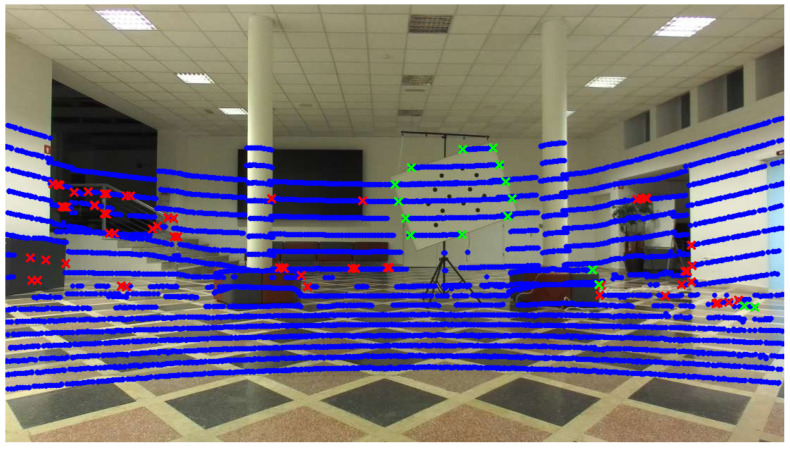
Calibration of the target edge candidates in the LiDAR point cloud. All the LiDAR points are shown in blue, the raw line segment edge points obtained by Algorithm 1 are marked with red, while the distance-filtered edge points are depicted in green. Only the points marked in green are used for the camera– LiDAR calibration.

The calibration target edges need to be detected in the LiDAR point cloud as well. This is performed by extracting planar line segments. These are subsets of the point cloud that lie on the same 3D line and correspond to a planar surface in the observed scene. Since the calibration target is planar, the starting and ending points of these segments correspond with the edges of the target object, as shown in Figure 5. The process of extracting these stating and ending points is described in Algorithm 1. It does not explicitly extract the points that lie specifically on the target plane edges, but it produces a sparse set of edge points that also include the edges of the target plane. The algorithm requires a threshold *t* that determines the maximum 3D distance between the points that can be included in the same line segment. If a new point is significantly distanced from the previous point, either due to a large depth disparity or missing LiDAR returns, the segment is closed, and a new segment is created. The algorithm works under the assumption that the points are sorted sequentially (i.e., by the LiDAR azimuth angle) and that the points produced by the same LiDAR beam are grouped together. This simplifies a 3D space line fitting problem to a simple linear search with a 3D collinearity check. The function isCollinear uses an implied distance threshold to account for the sensor noise; so, the points are not required to be strictly collinear but simply within an error margin. The candidate edge points are subsequently filtered using the target distance, i.e., points significantly farther or closer to the camera than the target are ignored. The approximate target distance can be obtained from perspective-n-point methods such as EPnP [13]. The results of the LiDAR line segment extraction are shown in Figure 5.
**Algorithm 1:** LiDAR planar segments detection algorithm**Require:** L = list of LiDAR beams
**Ensure:** S = list of line segments    S← [ ]    **for** beam in *L* **do**          segment← [ ]          first←NULL          last←NULL          **for** point in beam **do**                **if** empty (segment) **then**                     segment.insert (point)                      last←point                **else**                     **if** is Collinear (first, last, point) **and** $∥point,last∥<t$ **then**                           segment.insert (point)                     **else**                           *S*.insert (segment)                           segment← [ ]                           first←NULL                           last←NULL                     **end if**                **end if**          **end for**    **end for**


#### 4.2.3. Optimization of the Camera– LiDAR Transformation and Rotation Parameters

A well-calibrated camera–LiDAR pair is the one where the 3D points corresponding to the target edges lie on the target edges in the image, when they are projected onto the image plane using the camera’s intrinsic parameters in matrix K. If the camera’s intrinsic parameters are known, this will hold when we use a transformation matrix that transforms the LiDAR coordinate system to the camera coordinate system. Such a matrix consists of an axis convention transformation matrix C that transforms the axes of one coordinate system to another multiplied by a matrix composed of the relative rotation R and relative translation t between the two sensors (a 4×4 matrix M).

The matrices used are defined as follows:

(1) $$K=\left[\begin{array}{cccc} f_{x} & 0 & c_{x} & 0\\ 0 & f_{y} & c_{y} & 0\\ 0 & 0 & 1 & 0 \end{array}\right],\;M=\left[\begin{array}{cc} R & t\\ 0 & 1 \end{array}\right]=\left[\begin{array}{cccc} r_{1} & r_{2} & r_{3} & t_{x}\\ r_{4} & r_{5} & r_{6} & t_{y}\\ r_{7} & r_{8} & r_{9} & t_{z}\\ 0 & 0 & 0 & 1 \end{array}\right],\;C=\left[\begin{array}{cccc} 1 & 0 & 0 & 0\\ 0 & 0 & -1 & 0\\ 0 & 1 & 0 & 0\\ 0 & 0 & 0 & 1 \end{array}\right].$$

**Figure 6:**
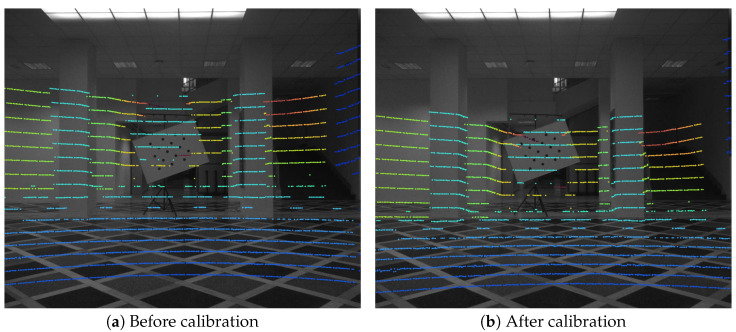
LiDAR points projected onto the polarization camera image. The color map depicts the point distance, with the blue-hued points being the closest to the camera and the orange-hued points being the furthest from the camera.

Since the axis convention transformation matrix C is known from the sensor specification, only the relative rotation and translation need to be estimated. If a set of LiDAR edge points is projected onto an image using the incorrect rotation and translation values, the LiDAR points’ projections and target edges in the image will not be aligned (see Figure 6a).

The set of extrinsic parameters is represented as:

(2) $$\Omega =[t_{x},t_{y},t_{z},\vartheta ,\varphi ,\psi ],$$

where the Euler angles that describe the rotation about each of the three axes are ϑ, φ, and ψ, which represent the pitch, roll, and yaw, respectively, and $t_{x}$, $t_{y}$, and $t_{z}$ are the translation parameters. In order to evaluate the cost function, the rotation matrix R is constructed as follows:

(3) $$R=R_{z}\left(\psi \right)\;\cdot \;R_{y}\left(\varphi \right)\;\cdot \;R_{x}\left(\vartheta \right),$$

where $R_{z}$, $R_{x}$, and $R_{y}$ are functions that construct a 3×3 rotation matrix that performs the rotation for the given angle on the corresponding axis. The elements of Ω are used to construct the rotation matrix R and the translation vector t and are combined into the transformation matrix $M_{\Omega }$. The projection of LiDAR points to the image is performed as follows:

(4) $$p=\Theta \left(P,\Omega \right)=K\;\cdot \;M_{\Omega }\;\cdot \;C\;\cdot \;P,\;P=\left[\begin{array}{c} X\\ Y\\ Z\\ 1 \end{array}\right],\;p=\left[\begin{array}{c} u\\ v\\ 1 \end{array}\right],$$

where *P* is the point in 3D space and *p* the projected 2D point. $M_{\Omega }$ denotes the matrix M constructed using a set of extrinsic parameters Ω, and Θ(·,·) represents the 3D to 2D projection of a point using a vector of extrinsic parameters. The matrix C is the axis convention transformation matrix, as defined in (1).

To enable automatic optimization, a cost function measuring the correctness of the current parameter set must be used. Given the extracted image and LiDAR features, as described in Section 4.2.1 and Section 4.2.2, the cost function *L* can be formulated as:

(5) $$L\left(I,P,\Omega \right)=\sum_{i=0}^{N}I_{f}\left(\Theta \left(P_{i},\Omega \right)\right),$$

where *N* is the number of projected LiDAR points, $I_{f}$ is the image of the smoothed target edges (image features, as shown in Figure 4d), *P* is the set of LiDAR target-edge points, and Ω is a vector of extrinsic parameters. Since the image $I_{f}$ is the image of the target edges that was smoothed by a Gaussian filter, the contribution of poorly aligned points should be close to zero, and a strong gradient should exist near the solution with the peak at the exact edge location. A standard gradient descent approach is used to optimize the extrinsic parameters with respect to the cost function.

Since the cost function cannot be analytically derived, a numerical derivative estimation is used instead. A numerical derivative has to be calculated for each of the parameters at each optimization step. We use a central difference formula that approximates the derivative of our cost function as follows:

(6) $$L^{\prime }\left(I,P,\Omega \right)\approx \left[\frac{L\left(I,P,\Omega_{x+h}\right)-L\left(I,P,\Omega_{x-h}\right)}{2h}\right];x\in \Omega ,$$

where *h* is the step size, and *L* is the cost function. The notation $\Omega_{x}$ means that only the value of a specific element of Ω is changed. Thus, a vector of partial derivatives with respect to each of the extrinsic parameters in Ω is calculated. Therefore, the cost function is evaluated twice for each extrinsic parameter. The gradient is then used to update the solution. The initial values of Ω are obtained by measuring the physical distances of the sensors.

#### 4.2.4. Parameter Ambiguity

There exists a degree of ambiguity when solving for the optimal parameter set Ω. This means that small differentials in different parameters from the set Ω produce the same geometric (and visual) effect.

For example, the small change in translation $\delta t_{x}$ along the *x* axis has the same effect as a small change δψ in yaw ψ. The same is true for a small change in translation $\delta t_{y}$ along the *y* axis and a small change δφ in pitch φ.

As a consequence, using a single target image is a poorly conditioned problem, and the parameter set Ω may converge to an incorrect solution. To rectify that, a data set with samples that contain various target positions and corresponding LiDAR point clouds needs to be used. In this context, a single data sample denotes a camera image and a synchronized LiDAR point cloud.

The data samples need to include different positions of the target relative to the camera, especially with respect to the distance, since the effect of translation on the projection is reduced with the distance. Since the solution must hold for every data sample, the gradient should be computed for each of them at every optimization step. The gradients could then be averaged before updating the solution. However, this is computationally expensive, since the cost function needs to be evaluated twice for every parameter as per Equation (6). Instead, at each step of the optimization process, a randomly chosen data sample is used to calculate the gradient. This results in the cost function values changing less smoothly, but in our experience, it leads to a good solution as well. A small step size must be used for updating the solution to allow a consensus between data points and thus convergence to a stable solution.

#### 4.2.5. Focal Length

While estimating the intrinsic parameters has long been a solved problem in theory [35], problems with estimating focal length can occur when dealing in practice with more exotic cameras and lenses such as IR or thermal cameras. Specifically, since the focal length estimation relies on the observed scale of the calibration pattern scaling, it sometimes happens that the focal length is estimated poorly, and the residual error is incorrectly compensated by the algorithm by adjusting the distortion parameters (which, as a consequence, are then also poorly estimated). If such intrinsic parameters are used in the extrinsic calibration process, the resulting extrinsic parameters do not provide a correct mapping to the image space. When projecting LiDAR points onto an image, the only two parameters that affect the scale are the camera focal length *f* and the $t_{z}$ offset between the coordinate systems of both sensors. Thus, if the focal length is estimated incorrectly, the offset $t_{z}$ along the *z* axis needs to compensate for the scaling. This can lead to incorrect relative sensor position estimation and in turn to incorrect inter-camera alignment.

This problem can be solved by accurately measuring the physical positioning of the sensors along the *z* axis and fixing the $t_{z}$ axis offset during optimization. In theory, the origin of the camera coordinate system is not visible to the user, but in practice, at least in self-driving vehicles, the state-of-the-art cameras are small enough (centimeters), that even the approximate position of the origin is sufficiently accurate in comparison to the observed objects (calibration targets or otherwise), which are meters away.

This solution requires that the camera focal length *f* be included in the calibration process, since the projection scaling needs to be optimized. The focal length *f* of the camera is added as another parameter and included in the optimization process in exactly the same way as the extrinsic parameters. Fixing $t_{z}$ and adding *f* into the optimization process yields a modified parameter set for optimization, which is $\Omega =[t_{x},t_{y},f,\vartheta ,\varphi ,\psi ]$.

### 4.3. Camera–Camera Alignment

The precise estimation of the transformation between the coordinate systems of two or multiple cameras is generally used for well-defined setups, such as stereo systems, and within the pipeline of Structure from motion (SFM) or Simultaneous localization and mapping (SLAM) approaches. Such estimation is primarily conducted through the matching of appropriate features between two or more images.

In a stereo setup, the established procedure is to estimate the relative camera position and orientation by observing a known object using both cameras. By matching available object feature points in both cameras, the essential matrix *E* of the stereo system can be calculated, and together with calibration matrices $K_{l}$ and $K_{r}$ belonging to the left and right camera, the fundamental matrix of the stereo system *F* can be derived.

However, this procedure may be not sufficiently accurate in practice, especially when we remove the usual implicit constraint of a stereo system, which is that two cameras of the same modality, build, and characteristics are used. Differing resolutions, modalities, and aspect ratios, which are introduced into the problem by using heterogeneous cameras, require a different approach, which we propose below.

In autonomous driving, LiDAR is part of many successful sensor setups, due to its unique abilities to provide reliable and accurate 3D measurements, and the camera–LiDAR calibration has been described in the previous section. Therefore, the natural extension of our approach is to employ the camera–LiDAR calibration as a proxy for the camera–camera calibration as well. In this case, the transformation between two camera coordinate systems can be obtained without the need for direct feature extraction and matching, which might be prohibitively difficult for some less frequently used modalities (thermal and near infrared).

LiDAR also provides absolute distance measurements, and by using these, the quality of the camera–LiDAR calibration is expected to be higher than if only calibration target position estimations were used, which can be inaccurate at larger distances due to image noise and numerical errors.

Given the existing camera–LiDAR calibrations obtained by the method described in Section 4.2, establishing a pixel mapping between two cameras is performed as follows. Let $C_{1}$ and $C_{2}$ be coordinate systems of two arbitrary cameras in our sensor system and *L* be the LiDAR coordinate system. We use the notation ${}^{A}H_{B}$ to denote the transformation from coordinate system *A* to coordinate system *B* using a rigid transformation in 3D. Using the camera–LiDAR calibration procedure, we obtain transformations ${}^{L}H_{C_{1}}$ and ${}^{L}H_{C_{2}}$. The relative position between $C_{1}$ and $C_{2}$ can be established as follows:

(7) $${}^{C_{1}}H_{C_{2}}={}^{L}H_{C_{2}}{\left({}^{L}H_{C_{1}}\right)}^{-1},$$

denoting the transformation from coordinate system $C_{1}$ to the LiDAR coordinate system and then a subsequent transformation to coordinate system $C_{2}$.

Using the calculated transformation matrix ${}^{C_{1}}H_{C_{2}}$, we wish to align the pixels corresponding to the same points in the scene in both images. This process is generally referred to as image alignment or registration. If the scene observed with both cameras is at an infinite distance, the pixel mapping can be performed using homography. However, if the points *P* in the scene are not at an infinite distance from the cameras, the homography mapping will result in errors due to the parallax effect. The magnitude of the parallax-caused error increases with the physical distance between the sensors and is inversely proportional to the distance to the observed scene point.

**Figure 7:**
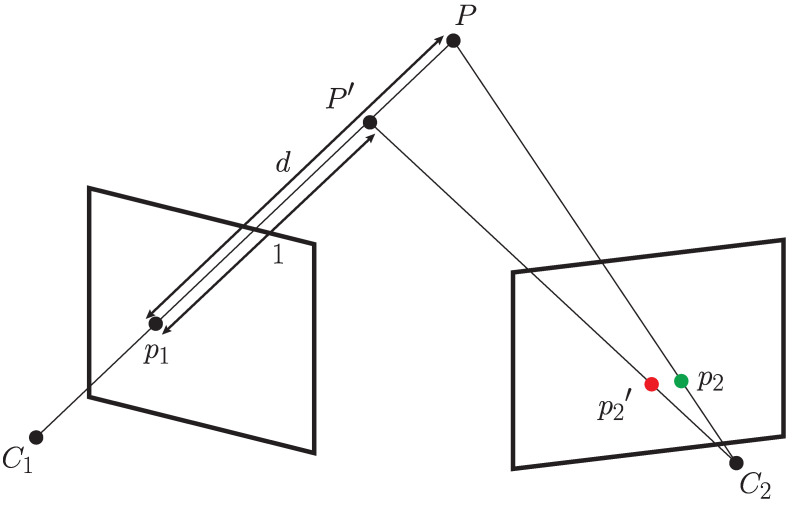
Depiction of the image alignment process. An existing point projection $p_{1}$ of point *P* is transformed into a 3D point $P^{\prime }$ using a depth value and the calibration parameters of $C_{1}$. If the depth value is incorrect, the transformation will result in an incorrect point, and its projection to $C_{2}$ will produce ${p_{2}}^{\prime }$, shown in red. If the correct depth is used, $P^{\prime }$ will be equal to *P* and thus correctly projected to $p_{2}$, assuming the calibration between cameras is correct.

Therefore, ${}^{C_{1}}H_{C_{2}}$ alone is not enough for correct pixel mapping. The process of mapping one image to another in general is as follows: 3D points have to be produced from the pixels of image $I_{1}$, which can then be transformed to $C_{2}$ using the transformation ${}^{C_{1}}H_{C_{2}}$ and finally projected to the image plane of $I_{2}$. The 3D coordinates $\tilde{P}$ corresponding to pixels from image *I*, can be acquired as follows

(8) $$\tilde{P}=K_{1}^{-1}p:p\in I_{1},$$

where $K_{1}$ is the calibration matrix of the first camera. This is geometrically equivalent to following the rays passing from the origin of the camera coordinate system through each of the pixel coordinates to an arbitrary distance. If the distance is set to 1, this procedure is sometimes referred to as the normalized plane (c.f. [11]). To correctly reconstruct the scene, a depth scalar needs to be applied to each of the points in $\tilde{P}$; then, the image mapping can be performed correctly as shown in Figure 7. Note that the mapping can be correct only for points whose depth corresponds to the actual depth of physical points in the scene, to prevent the parallax effect. The depth for each pixel from $I_{1}$ should be estimated as correctly as possible to produce a good mapping. Using an RGB-D camera solves this problem for closer ranges, but accurate depth information is not always available for all pixels, especially if they represent the projections of 3D scene points that are beyond the range of the depth sensor.

This process can also create some occlusion on closer objects, since some pixels in $I_{1}$ may not be visible from the viewpoint of $C_{2}$ and vice versa. If the estimated transformation of the camera coordinate system is correct, the pixel mapping error depends only on the quality of depth estimation. Any sensor that measures absolute depth can be used to correctly map the pixels. For far away points, even a rough estimation of the depth values can produce satisfactory results, as the parallax effect is reduced with distance. The same holds when mapping continuous regions of images, such as object detection masks or semantic labels.

## 5. Intermodal Annotation Transfer

Aligning images of multiple cameras of differing modalities can be used in a wide variety of scenarios, from extracting richer multimodal features to enabling object detection or classification under difficult circumstances (e.g., poor lighting, glare, etc.). However, if the data from different modalities are to be used in some sort of supervised learning, usually, at least some annotations have to be obtained. Since we are dealing with a multimodal system, the difficulty of this task increases with each added sensor or camera. The purpose of automatic full calibration is to correctly establish the relative positions of all the sensors and to subsequently allow data alignment for any arbitrary pair of sensors. Additionally, this approach can eliminate the need for annotating each modality independently, instead allowing for reusing existing annotations in one sensor to annotate data from the others. Finally, some modalities may not exhibit enough human-visible information for proper annotation, and intermodal annotation transfer is a necessity. This problem is, for example, the annotation of objects, whose highly distinctive feature is a color, in an infrared or thermal image, which do not preserve colors.

The benefits of only annotating one modality are manifold. Firstly, it requires only one pass over the data, and annotators can use rich RGB data that are easy to interpret for humans. No consolidation of multiple annotations of the same scene is required. This approach also enables the training of semantic segmentation models on each modality separately, so modality-specific models (e.g., thermal camera images for low-light navigation) can be trained. By the virtue of our camera–LiDAR calibration method, LiDAR data are also readily available as an additional data channel for every modality. If several modalities are overlaid onto a common image plane, a multispectral image can be constructed, which can help a feature extractor generalize better for single modalities as well as allow the entire model to exploit all the information provided by multiple modalities. Additionally, some modalities can be very difficult for human annotators to label, due to their unnatural appearance, lack of texture, or low resolution. This can be avoided by labeling only RGB images.

**Figure 8:**
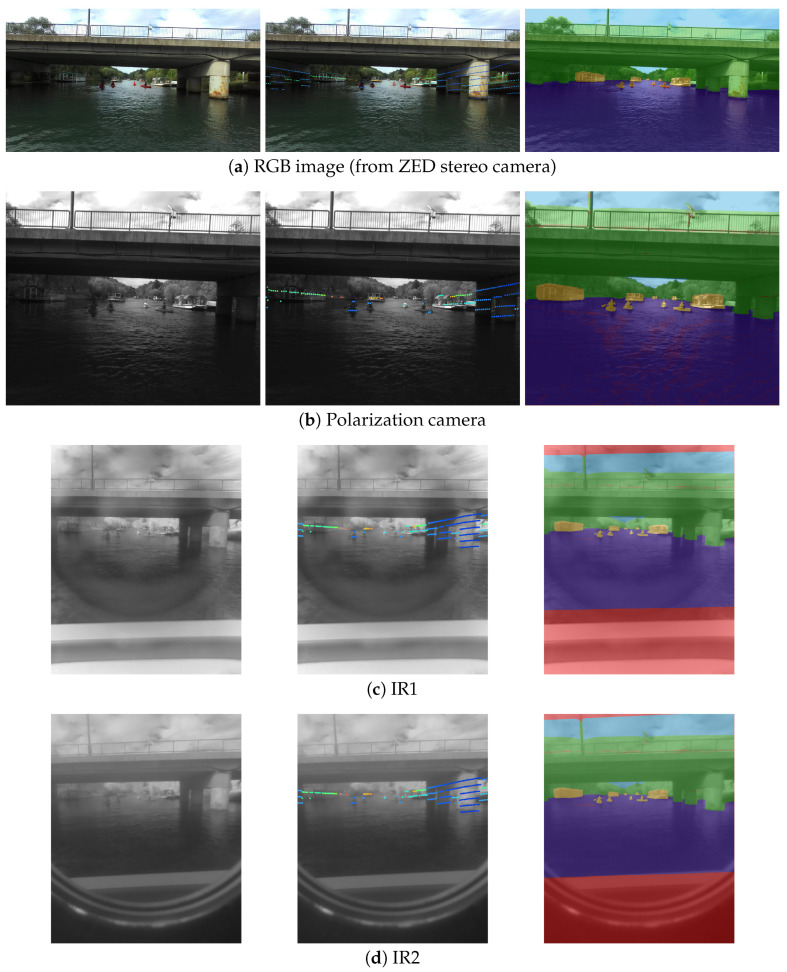

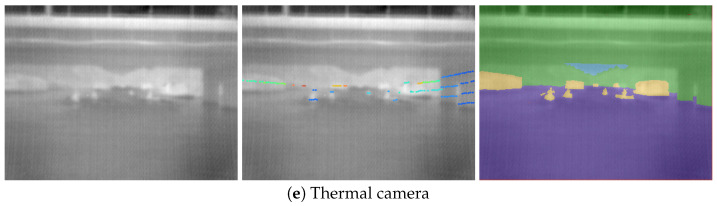
LiDAR points’ projection (central column, multicolor dots) and remapping (intermodal transfer) of the manual annotations (rightmost column, colored areas). The first column shows the raw images from different cameras. The middle column displays how the LiDAR points are projected onto each image. The last column depicts the transferred manual annotations, obtained originally in RGB images, onto other camera images and modalities. The pixels that have no source data are marked with red. Note that the number of LiDAR points is relatively low, as we are using 16-beam LiDAR, and LiDAR does not reflect well from the water surface.

To show the value of manual annotation transfer, we annotated a set of RGB images captured by our system and propagated them to other available modalities. The full results of our approach are shown in Figure 8. Only the labels shown in the rightmost image on the first row were produced manually, whereas the others were transferred using our approach. The images were labeled with the following categories: *sky*, *water*, *static obstacle*, *dynamic obstacle*, and *ignore*. These are the most pertinent categories for both obstacle detection and accurate detection of the water surface, which are most important for safe navigation. The category *static obstacle* in this case signifies the entire shore as well as piers, walkways, and moored boats or platforms.

### Obtaining the Pixel Depth

As described in Section 4.3, depth information is needed to correctly establish the pixel-wise mapping from one camera to another, even if the transformation of their coordinate systems are known. The depth data available for our system comes from two sources: the ZED stereo camera and the LiDAR sensor. Using this additional information can provide enough depth information to implement the label mapping in a way that is useful for other modalities. We used the left camera from the ZED system for the manual annotation due to its high resolution and wide viewing angle. In order to produce a good mapping, the depth has to be estimated at every pixel of the annotated image. The ZED stereo system is able to estimate the depth for a part of the scene up to 20 m away. While the LiDAR has a significantly larger range (up to 100 m), its data are much sparser even at close distances and are even sparser further away.

Our approach is to use the all the absolute depth measurements in a combined manner and then interpolate the depth values for which the depth measurements are not available. The depth interpolation needs to be anchored by the available measurements; thus, the interpolation anchor points are first sampled uniformly from the stereo depth data. The LiDAR points are then projected onto the image plane and added to the interpolation anchor points. Since the anchor points do not lie on a grid, a mesh-free method for interpolation is required, such as the RBF (radial basis function) interpolation [36]. The interpolated function is then evaluated at all pixels of the image, except for those covered by stereo data, which are dense, measured (not interpolated), and thus of superior quality. If necessary, additional control points can be added to include label-specific information. For instance, the sky label can be assumed to lie infinitely far away, while the water label is a plane roughly perpendicular to the image plane. Additionally, if semantic labels are available, their borders can be used to produce a higher quality depth estimation. The interpolation process tries to generate a smooth transition between anchor points, but this approach usually does not perform well when the neighboring anchor points belong to different objects or semantic labels. Depth interpolation can thus be performed on a semantic instance level, where only the pixels belonging to an instance are used to anchor the depth interpolation. When the instance level interpolations are merged, the sharp edges between the semantic labels are retained, and the mapping of instance edges is improved.

**Figure 9:**
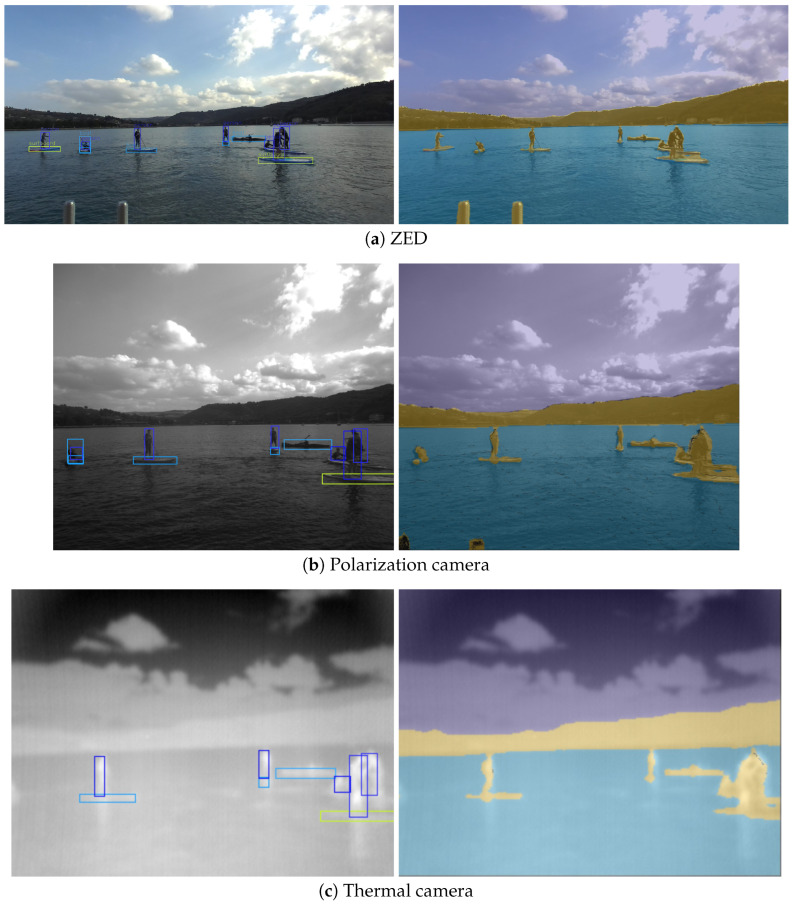
Transferring the detection and semantic segmentation results to secondary modalities. The left column shows the results from Yolov7 [37], while the right column shows the semantic segmentation results generated by WaSR [38]. Note that detection was performed on the images from the first row only, and rows (**b**,**c**) only show the transferred results.

Similarly to semantic labels, other image data or results can also be mapped using our method. This can be used for algorithm result verification, for bootstrapping manual annotation, for rich feature extraction etc. Figure 9 depicts how bounding box detections and semantic label predictions can be mapped between different camera images using our method.

By applying Equations (7) and (8) to each pixel of the source image, a 3D point cloud can be generated from one camera image, and the points can be projected onto the image plane of another camera. Bidirectional pixel mapping between both images is thus established. This allows mapping of the semantic labels from the annotated images to other modality images, as well as enabling mapping secondary modality images onto the main image plane. In this manner, a synthetic multispectral image can be generated that contains different pixel-aligned modalities that can be interpreted by a supervised learning model.

## 6. Experiments

The evaluation of our methodology consists of two quantitative experiments. In addition to the qualitative observation of the camera–LiDAR calibration, we experimentally evaluated the reprojection error using our multimodal target. Finally, we manually annotated a subset of our private multimodal dataset and examined the quality of the intermodal label transfer as compared to the manual annotations.

### 6.1. Camera–LiDAR Calibration Error

The camera–LiDAR calibration results can only be shown qualitatively due to the lack of ground truth measurements. Projection of the LiDAR points after complete calibration can be observed in the central column of Figure 8.

### 6.2. Evaluation of the Reprojection Error

The experiment was performed by using our own sensor system and observing the same calibration target with pairs of cameras of differing modalities simultaneously. Since the ground truth for our setup was not available and related methods were not applicable due to different data modalities, we compared our approach for aligning images to the EPnP [13]. PnP methods, including EPnP, estimate the relative position of a known object to the camera, when the camera calibration is known. Therefore, the relative position of two cameras can be established if the reference object is observed by both cameras simultaneously. In the case of this experiment, the reference object was our calibration target.

There was a significant difference between our approach and the EPnP. In our approach, the relative position of both cameras from the camera pair was constant after calibration. The reprojection error then reflected the overall accuracy of the calibration. Differently from that, each run of the EPnP algorithm on an arbitrary pair of images yields a separate estimation of the relative position of both cameras in a camera pair. Comparison between our method and the EPnP is impossible, unless we fix the relative position of both cameras for all image pairs. This was achieved as follows: we calculated the relative camera position for all the different target positions and then averaged the resulting positions into a single relative position matrix for each of the used camera pairs.

The reference coordinate system for our method was the LiDAR coordinate system, since both cameras were previously calibrated with respect to the LiDAR sensor, as described in Section 4.2. The reference coordinate system for the EPnP was the coordinate system of the target, as follows directly from the EPnP algorithm. Equation (7) was used in both cases to calculate the relative position of both cameras.

When the calibration target was detected in both images simultaneously, the pixel mapping was established, as described in Section 4.3. Using the pixel mapping, the coordinates of the calibration target pattern were transferred to the second image, and the coordinate error was calculated as the mean absolute error (MAE) between the 2D coordinates of the target pattern. The resulting error can be interpreted as a measure of the calibration quality when the target is observed from different angles and at different distances. The depth values required for the pixel mapping were taken from the depth camera, when available. If not, the LiDAR points that were projected onto the target surface were used for the interpolation of the depth over the entire target surface. Sequences of images were captured with both the visible spectrum calibration target and with the IR diode calibration target in order to compare as many camera pairs as possible. Four pairs of cameras were used for the evaluation, as shown in Table 1. The frames for which the target was detected in both cameras were sorted by the calibration target distance. The distance ranges in which the target was detected in both cameras are dependent on the camera resolution and field of view. Due to the lower resolution of the thermal camera, the target was not consistently detected beyond 6 m.

**Figure 10:**
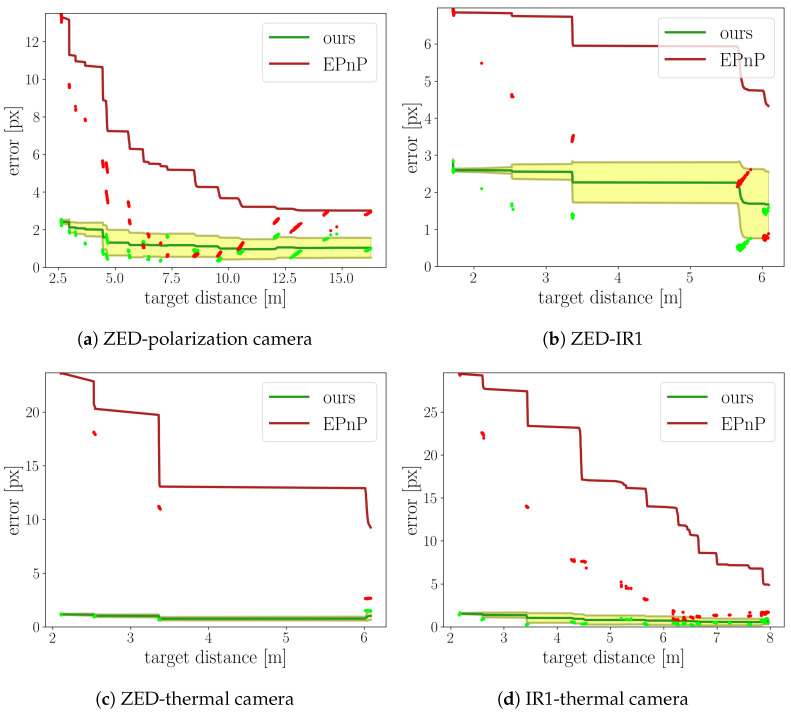
Reprojection error for the camera pairs. The raw error points for the EPnP and our method are depicted in red and light green, respectively. The cumulative average error is likewise shown in dark red and dark green. The shaded area represents the standard deviation of the error for our method.

The raw and cumulative error relative to the target distance is shown in Figure 10 for both approaches. It can be observed that the EPnP approach suffered from significantly higher raw errors at small distances, reaching a minimum at some point and then increasing further with the distance. This suggests that the EPnP has problems when a strong parallax effect is observed, while our approach does not.

The cumulative average error for both methods trended to lower values when the distance between the camera and the calibration target was increased; however, the values for the EPnP remained far higher than the cumulative average for our approach. The cumulative average was calculated using the formula ${CA}_{n+1}=\frac{x_{n+1}+n\;\cdot \;{CA}_{n}}{n+1}$. While the results of the EPnP approach were comparable to our method for some target distances, the error of the related method was dependent on the target distance and overall significantly higher than our proposed approach.

### 6.3. Evaluation of the Intermodal Label Transfer

**Figure 11:**
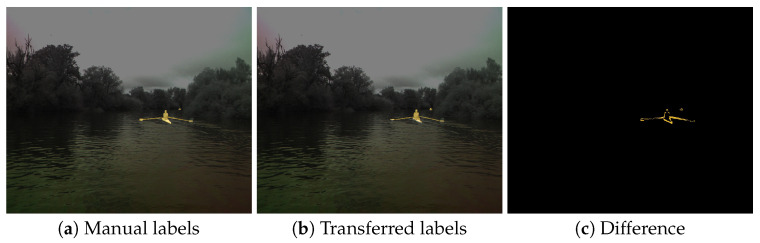
Comparison of the manual and transferred labels on the polarization camera images. The corresponding IoU is 0.49.

To further prove the utility of our approach, we manually annotated 15 images from each of the secondary modality cameras. Due to the high difficulty of annotating these images, we only annotated the *dynamic obstacle* class, as it is the most pertinent for obstacle detection as well as the easiest to manually annotate because of the clear borders between the objects and the background. The corresponding images from the ZED camera were also annotated; then, the annotations were transferred from ZED images to each of the secondary modality images. Comparison between the manual and transferred annotations was then performed by calculating the intersection over union (IoU) on all the image pixels. The average annotation transfer error for each camera modality is shown in Table 2. While the results were by no means perfect, given both the objective difficulty in annotating images captured on the water surface as well as annotating such different modalities, the results show the usability of our proposed method for transferring information between camera images in a multimodal sensor system. The difference in the average IoU values between different modalities was the consequence of those modalities being increasingly difficult to manually annotate due to the images’ visual appearance (see Figure 8). Nevertheless, following the long-established guidelines for object detection [39], which consider an IoU greater than 0.5 as a match, we claim that the results of our method are adequate and, most importantly, useful. This can be also seen in Figure 11—despite an IoU of 0.49, a very good alignment between the transferred annotation and manual annotation can be observed.

## 7. Conclusions

We presented a method that can be used for the efficient calibration of a variety of camera modalities with a LiDAR sensor. Additionally, a framework for using the resulting calibrations for establishing pixel mappings between multimodal images was proposed. Both can be used for automatic calibration of a multimodal system and enable the bidirectional pixel alignment of heterogeneous cameras. The resulting pixel mapping can then be used to facilitate manual or automatic image annotation with the purpose of multimodal deep learning for obstacle detection or semantic segmentation. Our future work includes publishing a multimodal fully annotated dataset captured by our platform, designed for joint supervised deep learning on vastly different modalities. Based on correct image alignment, training an existing detection or segmentation model along with additional channels could also be used to enable single-modality models to generalize better and improve their performance. Similarly, results from existing RGB-based methods could be used to automatically annotate different modalities and create single-modality datasets without the need for human annotation. Additionally, from semi-aligned camera data, multimodal monodepth-like models could be derived to estimate the missing depth data in order to further improve the pixel alignment between camera images. The main shortcoming of the presented approach is of course the need to have *some* kind of depth sensor present on an autonomous vehicle, be it LiDAR (for longer distance) or a stereo depth camera (for closer distances). However, in the domain of autonomous vehicles, this is not a huge burden, as it is accepted reality that either of those sensors is a must for navigation.

## Figures and Tables

**Table 1:** Camera–camera target pattern error per camera pair.

	Images	Distance Range [m]	$e_{\mathrm{EPnP}}$ [px]	$e_{\mathrm{ours}}$ [px]
ZED-polarization camera	654	3–16	3.02	1.03
ZED-IR1	385	2–6	4.33	1.67
ZED-thermal camera	112	2–6	9.26	1.09
IR1-thermal camera	554	2–8	4.90	0.63

**Table 2:** The average IoU error between the manual annotations and the annotations transferred from ZED images using our approach.

	$e_{\mathrm{IoU}}$
polarization camera	0.70
IR1	0.69
IR2	0.63
thermal camera	0.63

## Data Availability

Upon acceptance of the manuscript, all code and image data that are necessary for replicating the provided results will be provided at the following URL: (https://github.com/JonNatanael/multimodal_calibration, (accessed on 20 May 2023).

## Abbreviations

The following abbreviations are used in this manuscript:

FLIR	Forward-looking infrared
ICP	Iterative closest point
IoU	Intersection over union
LiDAR	Light detection and ranging
NIR	Near infrared
PnP	Perspective-n-point
RANSAC	Random sampling consensus
RGB	Red green blue
RGB-D	Red green blue depth
SFM	Structure from motion
SLAM	Simultaneous localization and mapping
